# Access to health care for Roma children in Central and Eastern Europe: findings from a qualitative study in Bulgaria

**DOI:** 10.1186/1475-9276-8-24

**Published:** 2009-06-30

**Authors:** Boika Rechel, Clare M Blackburn, Nick J Spencer, Bernd Rechel

**Affiliations:** 1 School of Medicine, Health Policy and Practice, University of East Anglia, UK; 2 School of Health and Social Sciences, University of Warwick, UK; 3 European Centre on Health of Societies in Transition, London School of Hygiene & Tropical Medicine, UK

## Abstract

**Background:**

Despite the attention the situation of the Roma in Central and Eastern Europe has received in the context of European Union enlargement, research on their access to health services is very limited, in particular with regard to child health services.

**Methods:**

50 qualitative in-depth interviews with users, providers and policy-makers concerned with child health services in Bulgaria, conducted in two villages, one town of 70,000 inhabitants, and the capital Sofia.

**Results:**

Our findings provide important empirical evidence on the range of barriers Roma children face when accessing health services. Among the most important barriers are poverty, administrative and geographical obstacles, low levels of parental education, and lack of ways to accommodate the cultural, linguistic and religious specifics of this population group.

**Conclusion:**

Our research illustrates the complexity of the problems the Roma face. Access to health care cannot be discussed in isolation from other problems this population group experiences, such as poverty, restricted access to education, and social exclusion.

## Background

Our paper reports findings on access to health services for Roma children that emerged from a wider qualitative study on access to child health services in Bulgaria in the context of the health care reforms that were implemented since the late 1990s. Similarly to other countries of Central and Eastern Europe, Bulgaria undertook substantial reforms of the financing, organization and delivery of its health services. These included introducing social health insurance and general practice, and reforming hospital services [1]. The impact of these changes on access to health services for children, particularly for disadvantaged groups such as ethnic minority children, has received hardly any attention so far [2].

Of the estimated 7–9 million Roma in Europe, most are living in Central and Eastern Europe, where they are estimated to account for over 8% of the population in Bulgaria, Macedonia, Slovakia and Romania (see Table 1) [3-5]. With a long history of discrimination and social exclusion, many Roma are reluctant to identify as such in censuses or other surveys that elucidate ethnic origin. Estimates of the number of Roma in different countries vary significantly, which is one of the challenges for epidemiological and health service research. According to the 2001 census results in Bulgaria, which relied on ethnic self-identification, the number of Roma in the country was 370,908, or 4.7% of the population. However, various other sources estimated the real number to be between 600,000 and 800,000, or 8–10% of the total population [6].

**Table 1:** Estimated number of Roma in selected countries of Central and Eastern Europe

Country	Number of Roma	Percent of total population [4]
Albania	90,000–100,000	2.6–2.9%
Bosnia and Herzegovina	40,000–50,000	1.0–1.3%
Bulgaria	700,000–800,000	8.3–9.5%
Croatia	30,000–40,000	0.7–0.9%
Czech Republic	250,000–300,000	2.4–2.9%
Hungary	550,000–600,000	5.4–5.8%
Macedonia	220,000–260,000	10.6–12.5%
Moldova	20,000–25,000	0.5–0.6%
Poland	50,000–60,000	0.1%
Romania	1,800,000–2,500,000	7.9–11.0%
Russia	220,000–400,000	1.5–2.7%
Serbia and Montenegro (including Kosovo)	400,000–450,000	3.8–4.3%
Slovakia	480,000–520,000	9.0–9.7%
Slovenia	8,000–10,000	0.4–0.5%
Ukraine	50,000–60,000	0.1%

Total	4,908,000–6,175,000	

Source: [5]

Note: Montenegro became independent from Serbia in 2006; Kosovo declared its independence from Serbia in 2008

The poor socio-economic status of Roma in Bulgaria and other countries in Central and Eastern Europe has been well documented. After the collapse of communism, the Roma were disproportionately affected by the hardship brought by economic transition. Low levels of education and lack of qualifications are some of the reasons for higher unemployment rates among the Roma. A 2001 survey in Stolipinovo, a suburb of Plovdiv, Bulgaria, which has about 40,000 inhabitants and is one of the largest Roma ghettos in Central and Eastern Europe, found that only 20% of respondents were in employment, and 53% had been continuously unemployed during the previous two years [7]. In Bulgaria 41% and in Romania 38% of Roma households were estimated to be poor in 2002, if the absolute poverty line was set at $2.15 purchasing power parity (PPP) per capita per day. These levels contrasted with 4% absolute poverty among non-Roma in Bulgaria and 7% in Romania. At the level of $4.30 PPP per capita per day, respectively 80% and 70% of the Roma in the two countries were estimated to be poor [8].

Maternal education has been shown to be an independent determinant of poor health outcomes in children [9]. In Bulgaria, enrolment rates in education show significant differences between the Roma and the majority population. Apart from being poor, Roma face further barriers in accessing education, including language barriers and geographic isolation. Roma are more likely to be enrolled in segregated schools or in "special schools" for mentally or physically disabled children, which limit future prospects of educational attainment and employment [10]. Segregated schools with predominantly Roma children have poorer infrastructure than the mainstream schools and lack materials and resources [10]. According to the 2001 census, the proportion of illiterate Roma in Bulgaria had increased to 18.1% [11].

While facing many of the same challenges, it is important to recognize that the Roma community in Bulgaria is characterized by considerable ethnic, linguistic, and religious diversity. In the 2001 census, 27.9% of self-identified Roma declared to be Muslims, while 86.2% gave Romani as their mother tongue [12]. Among the Muslim Roma, some speak Turkish as their first language, while there are also Roma who have Bulgarian as their mother tongue. These ethnic, religious and linguistic differences have implications for Roma health [13].

Despite their large numbers and the attention the situation of the Roma has received in the context of European Union (EU) enlargement, research on their health status and access to health services is very limited. Our study aims to start filling the gaps with regard to evidence on access to health services. It is one of the first qualitative investigations of access to health services for Roma children in Central and Eastern Europe and provides important empirical evidence on the complexity of the issues that need to be addressed.

A major challenge, but one which of necessity fell outside the scope of our qualitative investigation, is that, in many countries of the region, there is little statistical data disaggregated by ethnicity. The latter may be partly due to problems in recording and collecting health-related data according to ethnicity [14]. As a result, quantitative data on the health status of Roma in Bulgaria are scarce. The limited evidence that is available suggests that, throughout Central and Eastern Europe, life expectancy, infant mortality, and child and adult morbidity indicators are worse for Roma than for majority populations [15-18].

There are likely to be a number of reasons for the poorer health status of Roma. Throughout the region, they tend to occupy the lowest strata of society and face discrimination in all areas of life. Factors that impact on their health status include poverty, low levels of education, poor housing and sanitary conditions, and malnutrition. Poor access to health services is another likely contributing factor, but one which has not received much attention so far [19]. Our paper aims to extend the empirical evidence base on this question by exploring first-hand accounts of users, providers and policy-makers.

In conceptualizing the study, a broad view of access to care was adopted, recognizing the complex interaction of supply and demand side factors [20-22]. The concept of access to care describes the degree of responsiveness of service provision to users' actual needs, and encompasses availability of services (adequacy of supply with facilities, staff and other resources), physical accessibility, appropriateness and acceptability to users, and their affordability (relationship between cost of services and users' ability to pay) [23]. Further factors influencing access to care include information about health care choices and providers, education, cultural preferences and opportunity costs [22].

Confronted with diverse populations, all health systems are facing the challenge of ensuring equity and responsiveness to different population groups, including migrants, asylum-seekers, ethnic minorities and indigenous populations [24], and some of the lessons learnt from this can help to understand the situation of Roma in Central and Eastern Europe. In Australia, for example, meeting the health needs of its indigenous people, the Aborigines, has been described as one of the greatest challenges of the health care system [25]. Key policy propositions to deal with this challenge include an emphasis on primary health care, the importance of community control and participation, and an increased representation in the health care workforce [25]. In Central and Eastern Europe, as we have shown for the case of Bulgaria [26], people with disabilities continue to be confronted with enormous challenges, including high levels of institutionalization and prejudice, issues also faced by the Roma.

## Methods

The research reported in this paper formed part of a wider qualitative study on access to child health services in Bulgaria, based on in-depth interviews, a literature review and documentary analysis. Participants included policy-makers, health care providers, and users of services, from both rural and urban areas (two villages, one town of 70,000 inhabitants, and the capital Sofia). The inclusion of interviewees from a variety of backgrounds and settings, including not only users but also policy-makers and health care providers, aimed to increase the validity of our findings [27] and to gain a better understanding of the impact of health care reforms on access to child health services.

A total of 50 in-depth interviews were conducted by the lead author in Bulgarian and English. A topic guide, which differed for providers, policy-makers and users, was used as a flexible tool to guide the interviews and allow themes to emerge from the participants' accounts. Participants were recruited from the following groups:

• health care providers working with children (n = 13), including general practitioners, paediatricians, nurses and staff working in children's institutions;

• users of health services, i.e. parents and carers of young children from all four localities (n = 12), including Roma and parents of children with chronic illness or disability;

• policy-makers (n = 10), including representatives of the Ministry of Health, the Ministry of Labour and Social Protection, the National Health Insurance Fund, and international organizations;

• other stakeholders (n = 15), including NGOs working with Roma (4), NGOs working with institutionalized children (3), experts from academic institutions (5) working on Roma issues, institutionalized children or public health, a disability rights organization, a shelter for homeless youth, including young Roma people, and a social worker from a village with a large Roma population.

The interviews were conducted during four periods of field work between October 2005 and April 2007. All respondents were given detailed information about the study and informed consent to participate was obtained. The thematic analysis broadly followed the principles of grounded theory [28], to ensure that findings are "grounded" in the data retrieved, while recognizing that qualitative research is both inductive and deductive [29]. Ethical approval was obtained from the University of Warwick and from the Bulgarian Sociological Association.

## Results

Our study provides evidence that Roma children in Bulgaria face various barriers to accessing health services, including those related to poverty, lower levels of education, and living in geographically remote areas. Furthermore, Roma children and parents face discrimination and a lack of accommodation of their cultural, religious and linguistic differences.

### Poverty

As is the case for the general population [30], financial barriers experienced by parents of Roma children include formal and informal payments, costs of drugs and tests, and travel costs. These challenges are exacerbated by underdeveloped professional accountability and patient rights [1,30].

Members of the Roma minority tend to experience more difficulties in paying for health services, because many of them are unemployed and do not have any income. One council worker in a village with a majority of Roma inhabitants described the lack of job opportunities in the village:

" Most of them are unemployed. Actually everybody in the village is unemployed. Nowhere there is a source of living here, nowhere one could work. [...] The unemployment is the greatest social problem. Nobody has died of hunger, but everybody is living in poverty. One cannot say this is life, but they [the Roma] survive somehow with side incomes. If they earn five Lev today, they can survive today, and tomorrow is uncertain." (village council worker)

The poor economic situation of Roma affects their ability to access health services, due to the costs for travel and official co-payments for investigations and drugs. The resulting financial difficulties figured prominently in our interviews with Roma users. One Roma parent expressed her concerns about the lack of job opportunities and lack of money to provide food and clothing for the child and to buy medications in case of illness.

" The child benefits are little and only for one year: 18 Lev for one child per month. What can you buy with that? The child wants to eat. Very little money is given for the children. Earlier, the benefits were for two years, but now only for one year. Children's clothes are very expensive. Only shoes, such small shoes cost 7–8 Lev. There is no work. That's the bad thing in the village, that there is no work. I am unemployed. He is unemployed. He is on unemployment benefits now. He gets 36 Lev benefits and you are supposed to live on that. It is not enough for paying the electricity and water bills." (Roma user)

One of the Roma families we met had two children, one of whom had a recent episode of illness which started over the weekend. The family first went to the emergency services in the nearest town, and on the following day to see the general practitioner (GP). As there is no public transport, they asked fellow villagers to give them a lift with a car and paid 17 Lev (at the time of writing, 1 Lev was equivalent to 0.51 Euro) in total for the two journeys. The visits to the health facilities were free, but for the drugs they paid 23 Lev. The mother was employed through the social programme "From Benefits to Employment" and received the minimum salary of 160 Lev per month. Although health care at the point of use was free, the total cost of this treatment episode for the family (40 Lev for travel and medication) constitutes a significant part of their income. There are also services which require formal payments which Roma parents cannot afford:

" We also know that a number of health services in this country require additional payment, which children coming mostly from the Roma population are not able to pay. A lot of treatments are not covered by the Framework Contract between the Health Insurance Fund and the GPs. If you want a service which is not covered you have to pay this, which is legal." (international expert)

In addition, our respondents mentioned that physicians and nurses ask for informal payments from Roma patients, including for health services delivered to children:

" They don't have money for drugs either. Serious irregularities happen there, they commonly charge money for measuring their blood pressure, a 6 Lev fee. They charge money, for children also money is charged [...] For injections they charge regularly." (NGO representative)

In contrast, one of the interviewed Roma families did not recall paying for antenatal services or the delivery in hospital. Still, they gave gifts as a form of gratitude, illustrating the co-existence of informal payments for health care in Central and Eastern Europe in the form of both fee-for-service and gratitude payments:

Roma woman: "We did not pay anything. [...] Nothing. We only gave something to the doctors, out of gratitude."

Roma man: "Out of gratitude. We invited them. This is normal."

One policy-maker pointed out that financial barriers prevent Roma patients from going to see a doctor in the first place, because they anticipate they would not be able to pay for any investigations or drugs. Several participants mentioned that they had difficulties with the high cost of drugs, such as a Roma mother who contrasted the current situation with the previous child health system when medicines were free for children under three years of age:

" A week ago we bought linctus for cough. Only one drug costs 11 Lev something, only for the linctus. Previously, drugs for children used to be free. Until the age of three years, we received them for free. We got free prescriptions. However, now there aren't such prescriptions. If you have money, you buy the drug, if you don't, you leave it there. We borrow money from here, from there." (Roma user)

Lack of money was mentioned also as a main reason for the poor nutrition of infants and young children. Many respondents talked about the widespread practice to feed infants with yoghurt, rather than formula milk, because of financial difficulties. Among Roma, extreme poverty may mean that they cannot even afford buying cow's milk.

### Geographical barriers

Our respondents identified two major geographical barriers to accessing child health services for Roma: a poorer health infrastructure in rural areas and lack of health care providers (including emergency services) in Roma ghettoes in the large cities. One participant stressed that in villages there is no coverage with primary care providers on all days of the week. People also have difficulties going to town for diagnostic investigations, due to travel costs.

" There are serious problems for the people in the villages, not only Roma, but for all people, because the GP goes there once or twice a week. If tests need to be done, people have to go to the town, they have to spend money on transport. There is a big problem with the gynaecological examinations in the villages, mainly for the Roma. They simply do not go. They go twice, if they get pregnant and before giving birth just to register and that's it." (NGO representative)

The respondents also identified access to emergency services in Roma neighbourhoods as a major problem. Challenges mentioned include long distances from emergency care centres, poor-quality roads and discriminatory attitudes:

" In the Roma neighbourhoods, if there is an emergency, the ambulances refuse to go there. This is widely known. [...] There are two reasons. The one is that they really refuse to go there, and the other reason is that the ambulance cannot even enter the quarter and reach the address because of the infrastructure, narrow streets. There are such places." (NGO representative)

Our respondents believed that the most severe problems are experienced by the Roma who live in segregated areas or ghettoes, which in some places lack basic amenities, like running water, electricity and sanitation. One NGO representative drew attention to the fact that Roma who live among the rest of the Bulgarian population do not experience the same problems in accessing health services as those living in ghettoes. In contrast, children living in the ghettoes were believed to have limited access to primary care, because there are relatively few physicians working in the ghetto itself, or, if children are registered with a GP outside the ghetto, traveling to the GP when needed is problematic:

" In the ghettoes, in most cases the general practitioners who provide primary care and are supposed to be closest to the people are relatively few. Or if children are registered with such physicians, very often they are not in the ghetto itself. They are outside the ghetto, and the access to them is limited due to the distance – very often there is no possibility to travel and so on." (NGO representative)

### Low levels of education

Many participants mentioned the low level of education of Roma parents as a major barrier to accessing health services. As one NGO representative reasoned, better education is linked to basic health and hygiene habits, but education also influences health through better jobs and better living conditions. Several participants noted that education has deteriorated during recent years, affecting mostly young Roma and rural people, and resulting in increasing illiteracy.

" The level of illiteracy among the Roma population is extremely high. Nowadays, nearly 30–40 per cent of the young generation cannot read and write, and speak Bulgarian with great difficulty. And you could imagine that girl how often she would go to the doctor, if at all. If she asks a question, in what language one could explain to her. The information needs to be in a simple language using visual aids." (policy-maker)

### Administrative barriers

Many respondents identified administrative barriers for Roma children accessing health services. Several physicians and policy-makers mentioned that some Roma children are not registered with GP practices, despite their entitlement to free access to primary care regardless of the insurance status of their parents. According to the participants, difficulties in registration arise from changes in place of residence and lack of identity documents, problems that have been described in many countries of the region [18,31]. One GP described the case of a newborn Roma baby who she could not register or refer to a specialist, because the baby did not have an identity number. Lack of registration with a GP and lack of identity documents was identified as a barrier also for pregnant women who rarely attend antenatal care:

" Access to health care is impaired even before the child is born. The mother very often doesn't go to consultations at all, she is not registered anywhere. Very often there are people in these places who don't even have [identity] documents." (NGO representative)

Newborn babies in general are not automatically visited at home after discharge from maternity hospitals; rather a home visit requires the parents to contact their GP and request a visit. One hospital paediatrician expressed her concern that Roma children thus drop out of the system in the most critical period in life:

" The highest risk contingent are some of the minority groups, who exactly in that period drop out of the system. They have not arranged with a family physician [to visit the baby], they are not registered with a GP, they have [...] low levels of education, and actually these children, in their first month, are at highest risk, when the mortality is the highest too." (paediatrician, regional hospital)

### Health care reforms

In view of generally lower levels of education, higher illiteracy, and the fact that many Roma in Bulgaria have a mother tongue different from Bulgarian, accessing information about health services and the ongoing health care reforms, completing registration forms, or understanding information leaflets are a greater challenge for this group of the population. One NGO worker pointed out that information barriers are experienced by all people, but, due to their social and geographical isolation, the Roma are most affected by the general lack of information about health care reforms:

" Of course, the Roma remain the most isolated, because they are the least informed. They get least assistance to be informed." (NGO representative)

A common view on the impact of the health care reforms on access to care was that some of the introduced changes tended to further disadvantage the Roma. For example, the introduction of a health insurance system in the country in 1998 left disproportionately many Roma without entitlement to health care. Although this barrier is not applicable to children who are health-insured by the state, other reforms have impacted on Roma children's access to care, such as discontinuation of school health services, insufficient information about entitlements and official co-payments, and the need of being registered with a GP to access primary care, as discussed above.

### Discrimination

Participants described discriminatory attitudes on behalf of health care providers, as well as feelings of mistrust among the Roma. Discriminatory attitudes interfere with the process of seeking and receiving health services and constitute communication barriers between ethnic Bulgarian doctors and Roma patients. One participant noted that some GPs do not enroll Roma patients on their registers, as Roma are perceived as having more health problems.

" We are facing a situation that a number of GPs have a strong tendency not to accept clients coming for instance from the Roma minority, as these people have very often health deficits which are connected with their social status." (international expert)

Another participant described some of the problems and misunderstandings that arise in the interaction between (ethnic Bulgarian) physicians and Roma patients:

" In general it is very difficult to work with Roma. All physicians say that. Firstly because there are barriers and mistrust, and secondly because they cannot explain what the [health] problem is. And because they really seek help only in crisis situations, when it is in principle difficult to provide quality care. Of course there is, how to say it, I don't want to call it discriminatory attitude, but it is derogatory [...] They [the Roma] feel this attitude and of course they don't like to go there to be humiliated, as would every other human being." (NGO representative)

Although discriminatory attitudes may not always be present, stereotypes and anticipated discrimination impact on access to health services. As one participant explained, some Roma may not arrange an appointment beforehand and, if other patients are seen first, this may appear to them to be discrimination and result in negative feelings towards health care providers.

### Cultural differences

Many participants described cultural, linguistic and religious differences as barriers to accessing health services. An underlying reason is the very small number of Roma physicians and medical staff, leaving ethnic Bulgarians as the main providers of health services. To overcome the resulting barrier, between 2003 and 2007, up to 113 Roma health mediators were trained and certified, although many more would be needed [13].

Bulgarian is the only official language of the country and health services produce written information only in Bulgarian. Poor knowledge of Bulgarian among Roma can thus interfere with their ability to communicate with health care workers and to access health information. Other cultural barriers mentioned by our participants included religious beliefs, traditional remedies, the practice of early marriages, and the lower social position of women in Roma communities.

It is perhaps not surprising then that participants describe current health care provision as giving rise to a lack of trust among the Roma, with negative implications for seeking health care, attending prophylactic examinations and immunizations, and adherence to physicians' advice. One NGO representative described the lack of trust as a very common problem. She attributed it in part to the poor communication skills and lack of cultural awareness among health care workers.

A strong theme in the interviews was that of childhood immunizations, mirroring problems in immunization coverage for Roma children in other countries of the region [32]. Fears that immunizations may be harmful to the children or cause infertility were mentioned by several non-Roma participants. Lack of communication or sufficient explanation to the parents about common side effects of the immunizations seems to be one reason for these fears.

" [T]he problem with immunizations has not yet been overcome. Very often the Roma community is afraid of these immunizations. They believe that it is something directed against them only, that it is not good for the children. There are enormous fears, if after one immunization, the child gets ill. It is ill for a week. They link this with the immunization and that's true, but nobody has explained to them that it is a normal reaction and so on. And they don't go for the second immunization." (NGO representative)

Several policy-makers pointed to the need to take into consideration cultural differences when planning health programmes and delivering health services for ethnic communities:

" What I think is missing is a differential approach to working with the various ethnic groups. [...] This context needs to be known, so that the programmes of work in their communities acknowledge the specificity of the culture of this ethnic or population group." (policy-maker)

The need of quality education for all children and preventing early drop-out from school was emphasized by many participants as a key for improving health education, raising awareness about health risks and building trust in health services:

" What needs to be done by all means is that children and young people are kept in school and receive better quality education. The more educated they are, the later they will get married. She will not be 12 or 14 years old when she gives birth. She will have more knowledge and will know how to react in one particular situation or another." (social scientist)

## Discussion

No systematic research has been undertaken so far on access to health services for Roma children in Central and Eastern Europe and the findings presented here provide important empirical evidence on where to direct future research and policy interventions. While we have aimed to increase the validity of our findings through inclusion of a large number of different stakeholders from a variety of settings and backgrounds and including both users and health care providers and policy-makers, given the small sample size of our study and our qualitative research approach, our findings cannot be easily generalized to Roma throughout Central and Eastern Europe. However, our study has highlighted a number of issues that are likely to be of relevance far beyond the sample of interviewees.

Poverty emerged in our research as the most important barrier for Roma in accessing child health services. Roma often cannot afford to pay the fare for the public transport to the nearest health facility, for medical examinations, drugs or nutritious food [13]. Many Roma choose not to visit a doctor because they would not be able to pay for the prescribed drugs, and instead resort to self-treatment [17].

Geographical isolation exacerbates problems in accessing services. Roma neighbourhoods are usually situated at the outskirts of cities or in isolated rural areas with lack of public transport and infrastructure. In cities, Roma neighbourhoods are often characterised by slum-like conditions. The housing tends to be substandard and often lacks access to basic amenities such as running water, electricity, paved roads, public lighting and telephones. Accommodation is frequently dilapidated and overcrowded. Health care facilities are often located kilometres away and difficult to reach [12,33].

Compared to the general population, a higher proportion of Roma live in rural areas (about half, compared to 31% of the whole population). In the last decades, there has been a growing misbalance in health service provision between urban and rural areas (when measured in the number of primary care staff, doctors or hospital beds) [34]. Qualified medical staff is concentrated mainly in the cities, while many rural areas have no doctor. Because of poor road conditions, ambulances are often unable to reach geographically isolated areas in emergencies [17,33,35].

One of the major administrative barriers to accessing health services for Roma identified by our respondents is the lack of health insurance. Indeed, many Roma in Bulgaria are not registered with the unemployment bureaux and do not appear on the lists of recipients of social benefits. In Roma villages people often do not know what they have to do to qualify for non-contributory health insurance [31,34]. A nationally representative survey in 2007 found that almost one third of Roma women in Bulgaria did not have health insurance [13]. Despite the exemption of children from health insurance contributions [36], some Roma children are not registered with a GP. Although the children of our Roma respondents were registered with a GP, this does not seem to be a representative experience. According to a survey conducted by Gallup in 2000, 40.4% of Roma had no family doctor [34]. In the Roma ghetto Stolipinovo in Plovdiv in Bulgaria, about one third of the respondents in a survey in 2001 had not registered their children with a GP [7]. In the same survey, only 5% of Roma parents of children under 18 years said that they bring their children to prophylactic examinations. Nearly half of the parents visited the doctor only if the child was seriously ill, the main reason for which was that they could not afford to pay for medical services or medicines [7]. In some cases GPs have reportedly turned down requests by Roma to register for medical services with them [37]. This corresponds with findings from Romania, where the overwhelming majority of Roma interviewed in a survey stated that family doctors had refused to accept them on their rosters [31]. This illustrates how access to health services for Roma in Central and Eastern Europe is interlinked with the challenge of ensuring that their human rights are respected [3].

Discriminatory practices and negative attitudes of health staff have also been recorded from across Central and Eastern Europe. Communication and understanding between Roma and health professionals is often poor. In Bulgaria, the lack of public or private telephones in Roma neighbourhoods makes it difficult to contact medical services in case of emergency. There have been numerous reports in Sofia, Plovdiv, Sliven and other cities in Bulgaria of cases when emergency medical services have refused to send an ambulance to Roma neighbourhoods because it was "too dangerous" or there were allegedly no vehicles available [31,33,35,37].

Cultural factors, attitudes and beliefs are also important. In a 1995 survey in Bulgaria, 26% of Roma respondents admitted that if a family member became ill they did not take her to a doctor at all, but used a traditional "healer", magic, prayer or herbal remedies. In other cases, they delayed seeking medical help until all other methods had failed [38]. Delay in presenting to health services may be one of the reasons for the higher infant mortality among Roma. As mentioned by our (non-Roma) participants, some Roma refuse immunizations, due to beliefs that these may sterilize their children. A survey in 2007 found similar mistrust towards health care workers, in particular among elderly Roma, including beliefs that health care workers might want to harm Roma patients [13].

## Conclusion

Our study provides further evidence that Roma children experience significant barriers in accessing health services. They are more affected by financial and geographical barriers than their ethnic Bulgarian peers. In addition, access to health services for Roma children is hindered by cultural barriers and negative attitudes by health care workers. Our findings illustrate the complexity of the problems the Roma in Central and Eastern Europe face. Access to health care cannot be discussed in isolation from other problems this population group experiences. Unemployment and poverty are widespread and affect access to health care. The poor quality of education increases barriers to seeking and receiving information and impacts on health care seeking behaviour and communication with physicians. Access to proper housing and community infrastructures also have an impact on health and access to health services. Thus, strategies to improve access to health care for Roma children in Bulgaria and elsewhere need to adopt an integrated approach addressing the complexity of the needs of these children.

While concerns have been raised about the commitment of the Bulgarian government to improve the protection of its minorities [39,40], the country has formally embraced the principles of equity and accessibility in its national health policy. Health also forms one of the priority areas of the Decade for Roma Inclusion, an initiative that aims to achieve by 2015 the full integration of Roma in society in a number of countries in Central and Eastern Europe. Bulgaria has adopted a health strategy for ethnic minority children and, with funding from the EU and the Open Society Institute, has also implemented several projects aimed to improve Roma health. These include the training of health mediators, the setting up of mobile health clinics, and the training of health care workers working with Roma [33]. These are important first steps, but it is apparent that much more remains to be done to ensure that Roma children do not have worse access to health services than their ethnic Bulgarian peers. This will need to include comprehensive and regularly updated data on the health status of the Roma and their access to health services. It will be particularly important to take account of the ethnic and socio-economic diversity that exists among the Roma and to ensure adequate Roma participation and representation, including through involvement of Roma NGOs and informal leaders.

## Competing interests

The authors declare that they have no competing interests.

## Authors' contributions

BoR conceived of the study, carried out the fieldwork and drafted the manuscript. CB and NS participated in the planning of the study and the interpretation of results. BeR participated in the coordination of the study and helped to draft the manuscript. All authors read and approved the final manuscript.
